# Electrospun Polyethylene Terephthalate Nonwoven Reinforced Polypropylene Separator: Scalable Synthesis and Its Lithium Ion Battery Performance

**DOI:** 10.3390/polym10060574

**Published:** 2018-05-23

**Authors:** Haopeng Cai, Xing Tong, Kai Chen, Yafei Shen, Jiashun Wu, Yinyu Xiang, Zhao Wang, Junsheng Li

**Affiliations:** 1 School of Materials Science and Engineering, Wuhan University of Technology, Wuhan 430070, China; cai_haopeng@whut.edu.cn (H.C.); 18771994076@163.com (X.T.); whutwjs@163.com (J.W.); 2 Wuhan Jingce Electronic Technology Co., Ltd., Wuhan 430070, China; chenkai@wuhanjingce.com (K.C.); shenyafei@wuhanjingce.com (Y.S.); 3 School of Chemistry, Chemical Engineering and Life Sciences, Wuhan University of Technology, Wuhan 430070, China; 243869@whut.edu.cn; 4 State Key Laboratory of Advanced Technology for Materials Synthesis and Processing, Wuhan University of Technology, Wuhan 430070, China

**Keywords:** separator, electronspin, PET nonwoven, polypropylene, lithium ion battery

## Abstract

A novel polyethylene terephthalate nonwoven reinforced polypropylene composite separator (PET/PP) with high thermal stability and low thermal shrinkage characteristic is developed through a scalable production process. In the composite separator, the electronspun polyethylene terephthalate nonwoven layer improves the electrolyte affinity and can sustain as the barrier layer after the shutdown of the polypropylene layer. Due to its high ionic conductivity, the PET/PP separator shows an excellent discharge capacity. In addition, the superior thermal stability of the separator significantly enhances the safety performance of the separator. Considering the feasibility of the large-scale production of the PET/PP separator and its superior battery performance, we expect that the novel separator could be a promising alternative to the existing commercial separators.

## 1. Introduction

Due to its high capacity, low self-discharge rate, long cycle life, as well as the feasibility of controlling the discharge performance through the battery management system [1,2], advanced energy devices such as a lithium-ion battery (LIB) are considered as an attractive power source for a wide variety of applications ranging from consumer electronics to electric vehicles. However, compared to their counterparts such as the proton exchange membrane fuel cell [3,4], possible safety concerns arising from the shortage of the LIB have posed a great threat for the practical application of LIBs [5,6,7]. All the components of the battery should be carefully engineered to improve the safety performance of LIB [8].

The separator is an indispensable component of the LIB, which could retain electrolyte, transport ionic conductor carriers between the two electrodes, prevent the shortage between electrodes, and perform safe deactivation of the LIBs under overcharge, abnormal heating, or mechanical rupture conditions. Polyolefin separators, such as polypropylene (PP) and polyethylene (PE), have been widely used for LIBs because of their superior mechanical and chemical stability. However, polyolefin separators have poor wetting/retaining ability to the electrolyte solutions and will shrink or melt at elevated temperatures, which tends to cause an internal short-circuit [9,10,11,12,13,14]. Coating the polyolefin separators with ceramic powders [15,16,17,18,19,20,21] is the most frequently used approach to solve the aforementioned problems. However, the ceramic powders normally have poor combability with the separator and the formation of a physically stable ceramic coating normally relies on the use of polymer binders. The use of the binder would inevitably decrease the porosity of the coating layer and significantly increase the thickness of the separator, which is unfavorable for efficient ion transport through the separator [22,23,24,25]. Compositing the polyolefin separator with a second porous polymer layer is another way that has been demonstrated to successfully improve both the electrolyte wettability and thermal stability of the polyolefin separator. Compared to the ceramic modification of the polyolefin separator, the functionalization of polyolefin separator with a porous polymer layer is more advantageous due to the good affinity between the polymer and polyolefin separator [26,27,28,29].

The electrospinning technique is a fascinating fiber fabrication technique to produce nanofiber and has been applied to form nonwoven electrolytic membranes with high ionic conductivity and good electrochemical properties [30,31,32]. In this study, a composite separator with an electronspun microporous polyethylene terephthalate nonwoven (PET) top layer and a commercialized polypropylene membrane as matrix support is prepared with the electrospinning technique. The major advantages of the novel composite separator over the traditional separator can be summarized as follows: (1) The PET layer in the novel composite separator is a dual-functional layer, which improves both the mechanical stability and electrolyte affinity of the composite separator. In contrast, the mechanical reinforcing layer in most commercial polymeric composite separator doesn’t provide additional functionality; (2) In a conventional ceramic coated separator, the use of a binder material is necessary to maintain the integrality of the whole separator, which may sacrifice the ionic conductivity of the separator. As a comparison, the reinforcing PET layer and PP base layer in the novel composite separator have a high compatibility due to their polymeric nature and the formation of the composite separator does not rely on the use of binder. We show that the novel separator has excellent mechanical properties as well as superior electrochemical properties. The effect of the structure parameters (such as the fiber diameter) of the PET nonwoven layer on the mechanical, thermal, and electrochemical properties of the composite separator is carefully studied and optimized. The optimal composite separator obtained in this study shows significantly enhanced stability as well as improved discharge capacity compared to the pristine PP separator.

## 2. Materials and Methods

### 2.1. Preparation of Composite Separators

Polyethylene terephthalate (PET, *M*_W_ = 150,000) was bought from yuanfang shanghai company (Shanghai, China) and dried in a vacuum at 60 °C for 24 h before used. The commercialized PP separator (thickness, 20 μm) made by the dry process was kindly supplied from Huilong Co. Ltd. (Wuhan, China). The solvents were reagent grade and used as received.

To prepare the solution for electrospun PET membrane fabrication, various amounts of PET were dissolved in the mixture of trifluoroaceticacid/1,2-dichloroethane (8:2, *v*/*v*) under stirring at ambient temperature for 12 h. The concentration of PET (*w*/*v*) was 16%, 18%, 20%, and 22% respectively [33].

The electrospinning process was carried out with an electrostatic spinning machine (SS-2535H, Ucalery, Beijing, China) using a voltage of 9 kV and a flow rate of 0.19 mm min^−1^ at 25 °C. The distance between the needle tip and the collector was set to be 15 cm. The commercialized PP separator was fixed on the collector plate with tape to collect the electrospun PET fibrous. After 5 mins of spinning, the PP separator covered with nanofiber nonwoven was aired at room temperature for 48 h to obtain the composite separators. For convenience of expression, the composite separators will be represented by PET/PP-X, where X represents the concentration of the solution used for electronspinning.

### 2.2. Characterization of Composite Separator

The thickness of the composite separator was measured by thickness gauge (CH-1-S, Shanghai liuleng). The reported thickness was the average value from 5 individual measurements at different points of the separator. The morphology and the average diameter of nanofiber was characterized and measured by a scanning electron microscope (FE-SEM, S-4800, Hitachi, Tokyo, Japan). The tensile strength of the separator was determined by using a Tensile Tester (Hualong WDW-0.5, Shanghai, China). The sample used for the tensile strength test had a size of 50 × 10 mm and the elongation rate was set to be 5 mm min^−1^. The contact angle (CA) measurements were carried out by using a contact angle meter (JC2000D POWEREACH, Shanghai, China). The CA was determined by means of sessile drop method, using electrolyte liquid (1 M lithium hexafluorophosphate (LiPF_6_) dissolved in the mixture of ethylene carbonate (EC), dimethyl carbonate (DMC), and diethyl carbonate (DEC) (EC:DMC:DEC = 1:1:1, *v*/*v*/*v*) as testing liquids. An electrolyte droplet with a volume of 5 μL was used for each contact angle measurement. The electrolyte uptake of relevant separators was measured by soaking the separators in the electrolyte solution for 30 min. The weight of the separators was taken after removing the electrolyte remaining on the surface of separators with filter paper and the electrolyte uptake was calculated with the following equation:

(1) $$\mathrm{electrolyte}\;\mathrm{uptake}\left(\%\right)=\left(w-w_{0}\right)/w_{0}\times 100$$

where *W*_0_ (g) and *W* (g) are the weight of the dry and wet separators, respectively.

DSC scans were performed with a simultaneous TG-DSC system (STA449F3, NETZSCH, Bavaria, Germany) at the rate of 5 °C min^−1^ under nitrogen purge from 30 °C to 300 °C. TMA tests were performed on a TMA analyze (TMA202, NETZSCH, Bavaria, Germany) at a ramping rate of 5 °C min^−1^ under nitrogen purge from 30 °C to 300 °C, with a constant external tensile force of 0.02 N. Thermal shrinkage was tested by placing the relevant separators sandwiched by two glasses placed in an oven at various temperatures from 120 °C to 180 °C for 0.5 h, after that, dimensional changes of the separators were measured by the following equation:

(2) $$\mathrm{shrinkage}\left(\%\right)=\left(A_{i}-A_{f}\right)/A_{i}\times 100$$

where *A_i_* (cm^2^) and *A_f_* (cm^2^) are the initial and final area of the relevant separator respectively.

### 2.3. Electrochenmical Performance of Composite Separator

The composite separator and PP separator were punched into circular pieces with diameter 19.6 mm for characterization. The circular separators were sufficiently soaked in liquid electrolyte in an Ar–filled glove box. The electrolyte contained 1M LiPF_6_ in ethylene carbonate (EC)-ediethyl carbonate (DEC)-ethylmethyl carbonate (EMC) (*v*/*v*/*v* = 1/1/1, LDC3045I). The soaked separators were sandwiched between two stainless steel electrodes and assembled in a tightly sealed test cell. The ionic conductivity of the separators at various temperatures was determined by an AC impedence analysis using an electrochemical workstation (CHI604D, CH Instruments, Shanghai, China) over the frequency range of 0.1 HZ-100 KHZ and under an AC voltage of 5 mV. The conductivity of the separator was calculated from the following equation:

(3) σ=d /(R*S)

where σ (mS cm^−1^) is the ionic conductivity, d (cm) is the thickness of the composite separator, R (Ω) is the bulk resistance, and S (cm^2^) is the area of the electrode.

The composite separators for the electrochemical stability measurement were sandwiched between Lithium metal and stainless steel electrodes in a test cell. The electrochemical stability was then measured by using a liner sweep voltammetry from 2.5 V–7 V at 1 mV s^−1^. For charge/discharge and cyclability tests, the half-cell was assembled by sandwiching the separator between a lithium anode and a LiFePO_4_ cathode and then activated by filling with liquid electrolyte. The LiFePO_4_ cathode (Kejing Zhida Ltd. Co., Shenzhen, China) with an areal density of 1.27 mg cm^−2^ was prepared using its blend with acetylene black and PVDF binder (HSV 900, Arkema Ltd. Co., Colombes, France) at a ratio of 8:1:1 by weight. The charge/discharge and cyclability tests of cells were performed using battery test equipment (CT2001A, LAND Electronics, Wuhan, China). The discharge current densities were varied from 0.1 C to 1 C under a voltage range between 2.5 V and 4 V. The cells were cycled at a fixed charge/discharge current density of 0.5 C (1 C = 170 mAh g^−1^).

The interfacial resistance between the separator and Lithium metal electrode was carried out by electrochemical impedance spectroscopy (EIS) over the frequency range of 0.01 HZ–100 KHZ and under an amplitude of 5 mV.

## 3. Results and Discussion

### 3.1. Membrane Characteristics of the Composite Separators

**Figure 1:**
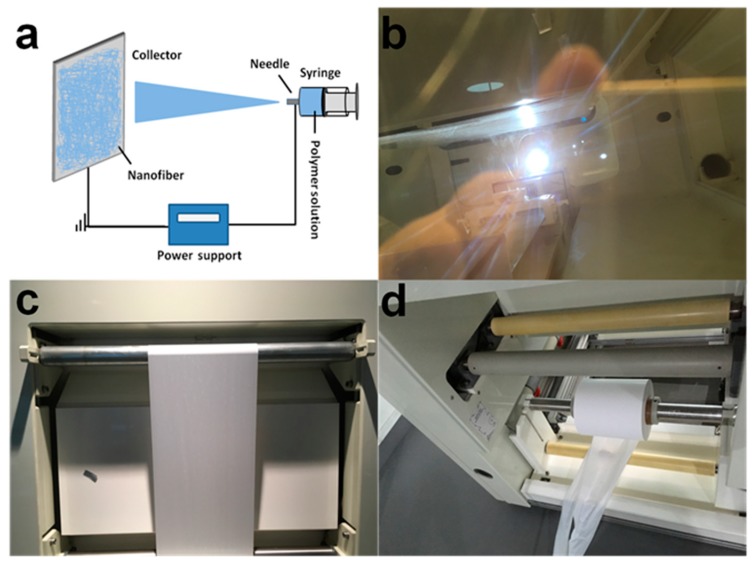
(**a**) Schematic illustration of the electrospinning; (**b**–**d**) photography of mass production for composite separator by free-liquid surface electrospinning.

**Figure 2:**
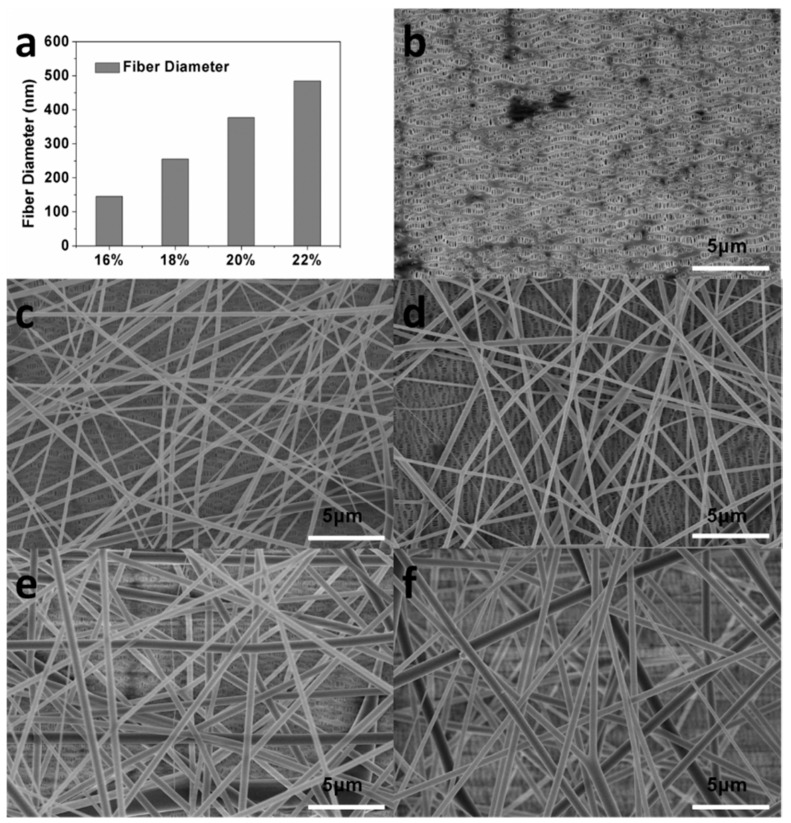
(**a**) The fiber diameter of polyethylene terephthalate (PET) nonwoven membrane top layer with different concentrations of PET; SEM image of the polypropylene (PP) separator (**b**) and composite separators (**c**–**f**): PET/PP-16%, PET/PP-18%, PET/PP-20%, PET/PP-22%.

As shown in Figure 1, our synthetic process allows for continuous and large-scale modification of the separators, which is critical for practical production of the separators. To investigate the effect of structural parameters of PET on the performance of the composite separator, PET nanofibers with different fiber diameters were prepared by varying the concentration of the solution used for electronspinning. The correlation between the diameter of the PET nanofiber and the concentration of the solution used for electronspinning is shown in Figure 2a. As shown in the figure, slightly varying the solution concentration could result in obvious change in the diameter of the obtained PET fiber. The SEM images of the pristine PP separator and PET/PP composite separator on the PET side was shown in Figure 2b–f. As can be seen, the PP separator exhibits typical slit pores with the pore size in the range of sub-micrometer. For the PET/PP separator, randomly oriented PET nanofibers were uniformly distributed and formed a three-dimensional porous network on the PP separator. The pore size of the PET nonwoven layer is significantly larger than that for the PP layer, which is beneficial for efficient ionic transportation across the separator.

**Figure 3:**
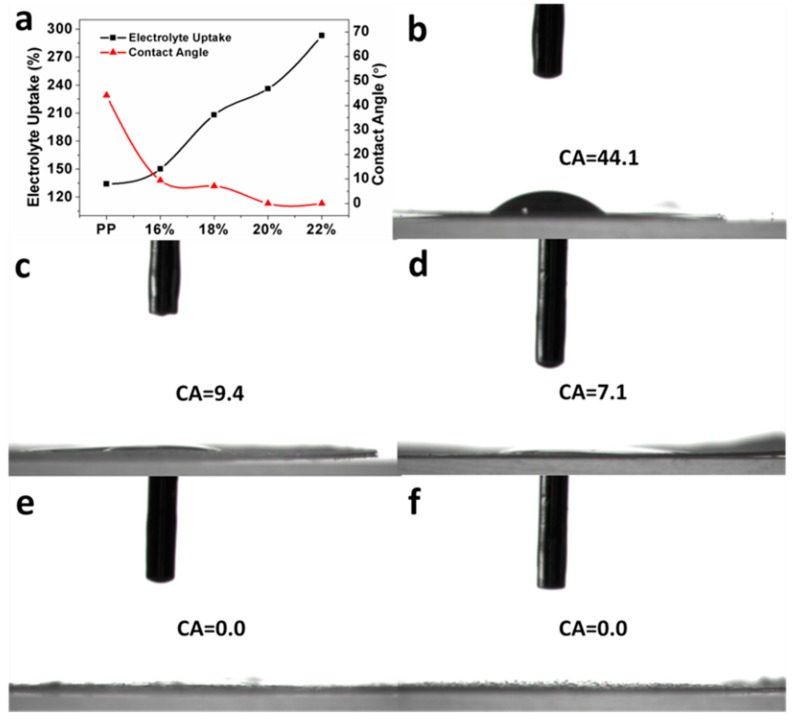
(**a**) The contact angle and electrolyte uptake of the PP separator and the composite separators; photography of the PP separator and the composite separators. Images showing the electrolyte droplet on (**b**) PP separator; (**c**) PET/PP-16%; (**d**) PET/PP-18%; (**e**) PET-PP-20%; and (**f**) PET/PP-22%.

The rapid and efficient wetting property of a separator toward typical battery electrolytes is important for the cell assembling process and its electrochemical performance. To quantitatively evaluate the electrolyte wetting properties of the separators, the static CAs of electrolyte on the separators were measured (Figure 3). The PP separator showed a high electrolyte contact angle of 44.1° due to its low polarity. In contrast, the composite separator showed improved electrolyte wettability. The static electrolyte contact angle for PET/PP-16%, PET/PP-18%, PET/PP-20%, and PET/PP-22% was measured to be 9.4°, 7.1°, 0°, and 0°, respectively. The decreased electrolyte contact angle of PET/PP separator is originated from the presence of polar functional group on the PET surface as well as the increased surface roughness brought by the PET nanofiber. The PET/PP separator with a larger fiber diameter had a higher roughness, and thus showed improved electrolyte wettability. The decrease in CA of the composite separator indicates that the modification of the PP separator with the PET nanofiber was an effective method to enhance the wettability of the PP separator. The electrolyte uptake capability of the separators was also quantified to further evaluate the electrolyte affinity of the separators. Generally, the separator with a lower electrolyte contact angle had a higher electrolyte uptake. The electrolyte uptake was as high as 293% for PET/PP-22%, much higher than the 134% for that of the pristine PP separator.

**Figure 4:**
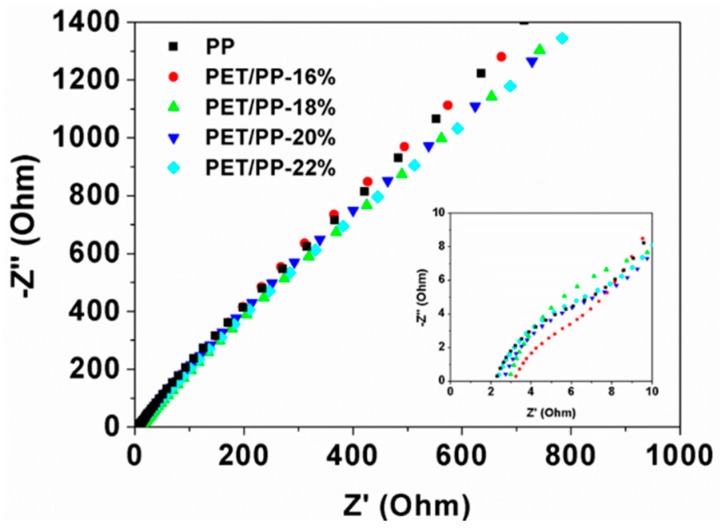
The AC impedance spectra of symmetric cells SS/separator/SS with PP separator and the PET/PP separators.

The conductivity of the separator was measured to evaluate its potential in LIB applications (Figure 4). Although the total ionic resistance of the PET/PP separators are slightly higher than the pristine PP separator, the calculated conductivity of the PET/PP separator is higher than the PP separator due to the increased thickness of the PET/PP separator (Table 1). PET/PP-22% showed the highest ionic conductivity of 0.782 mS cm*^−^*^1^ among all the separators. The high ionic conductivity of PET/PP separators can be correlated to their high electrolyte affinity.

**Figure 5:**
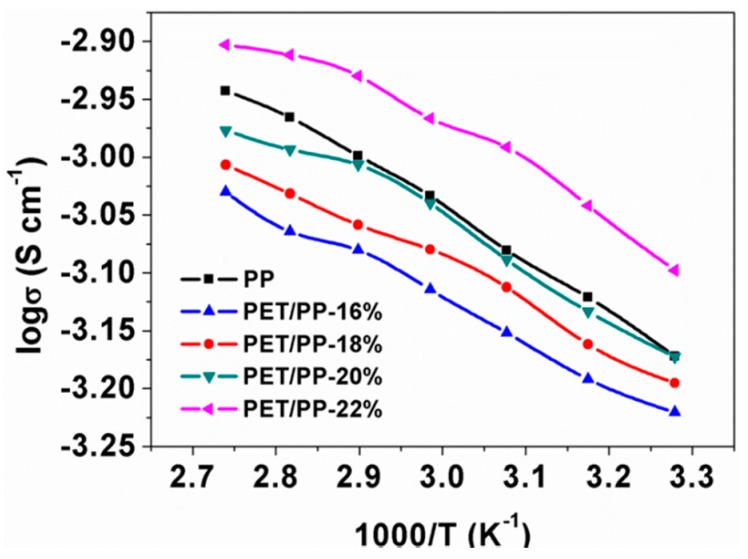
Temperature dependence of ionic conductivity of PP separator and the PET/PP separators.

Furthermore, the ionic conductivity of the separators was measured at different temperatures to investigate the potential of the separator at high temperature operation (Figure 5). The conductivity of the separators generally increased with the rise of the temperature. For the PP separator, its ionic conductivity increased from 0.67 mS cm^−1^ at 30 °C to 1.14 mS cm^−1^ at 90 °C. PET/PP-22% showed the highest ionic conductivity in the whole temperature tested. In addition, a good linear relationship was found between the logσ and T^−1^, suggesting an Arrhenius type kinetics for the ionic transport through the separator. The similar slope of the logσ vs. T^−1^ curves of the separators indicated that the incorporation of the PET layer onto PP didn’t increase the activation barrier of the ionic transport.

### 3.2. Mechanical and Thermal Properties of the Composite Separators

**Figure 6:**
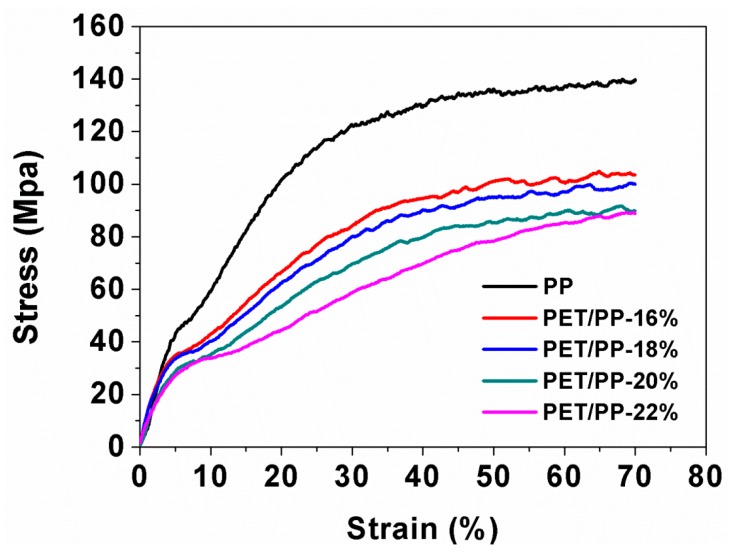
Stress–strain curves of PP separator and the PET/PP separators.

An applicable separator should also have excellent mechanical properties. The mechanical property of the separator was characterized by the stress–strain curves shown in Figure 6. It was previously reported that typical nonwoven PET nanofiber normally has a low tensile strength [34], which is because that the mechanical strength of the PET non-woven is mainly contributed by the intra-molecule interactions between the fibers. Due to the low tensile strength of the PET component, the PET/PP composite separator showed lower tensile strength than the PP separator and the tensile strength of the PET/PP separator decreased with the increase of the PET component in the composite separator. However, it should be noted that the PET/PP-22%, which exhibited the lowest mechanical performance, had a tensile strength of 85 MPa, which may be already high enough for practical battery applications [35]. In addition, the PET/PP separator had a high elongation at break, similar to that of PP separator.

**Figure 7:**
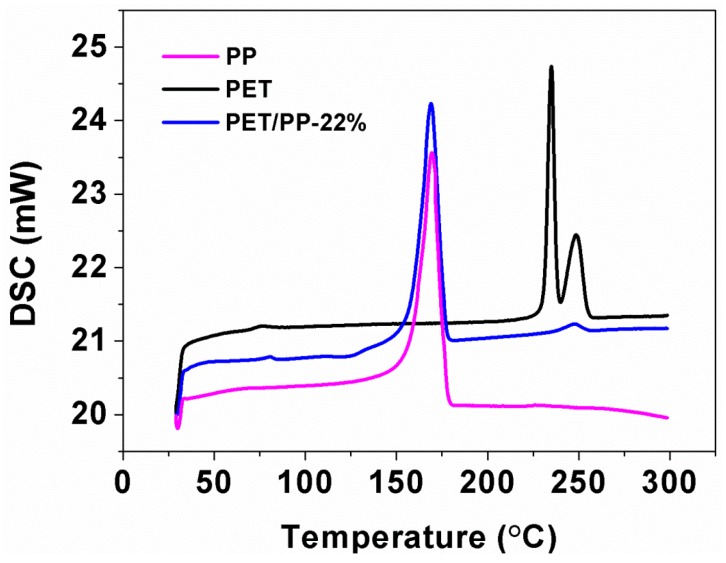
DSC curves of the PP separator, the PET/PP-22% and PET granular materials.

DSC curves of the PP separator, the composite separator and granular PET material were recorded to study the thermal stability of the separators (Figure 7). It is clearly observed that the PP separator gives a single endothermic peak at ~169 °C due to its melting. The PET/PP separator showed two endothermic peaks located at ~169 °C and ~248 °C, which can be assigned to the melting of PP and PET, respectively. One of the important advantages of the PET/PP separator is that the two-layer structure enables a thermal shutdown function that could be used to avoid thermal runaway in Li–ion batteries. For the PET/PP separator, the PP separator could thermally shutdown at ~169 °C under abnormal situations, while the PET nanofiber nonwoven membrane could maintain the integrity of the whole separator up to 248 °C, thus allowing for a safe operation of the battery.

**Figure 8:**
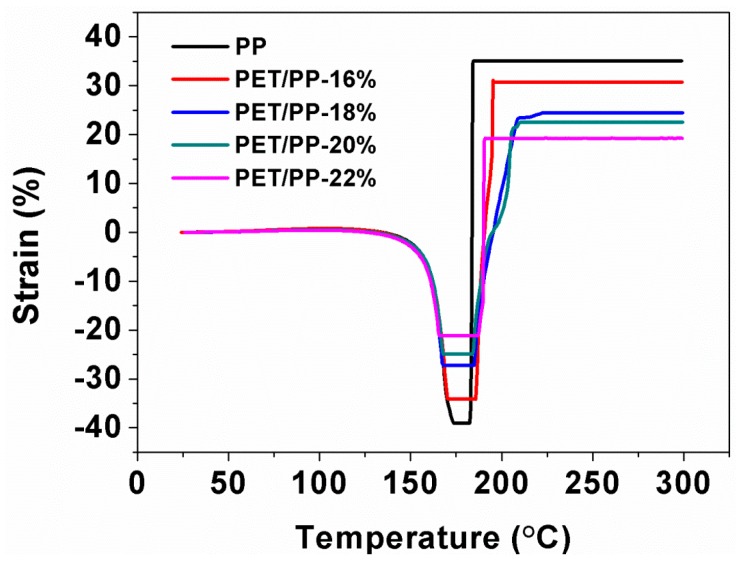
Thermomechanical behavior of the PP separator and PET/PP separators.

Figure 8 presents the dimensional changes of relevant separators as a function of temperature. As we can see from the TMA curves, a slightly lower degree of thermal shrinkage occurred with the PET/PP separator with a larger PET nanofiber. Compared to the PP separator, the maximum length reduction of the composite separator was far lower (22% vs. 40%).

**Figure 9:**
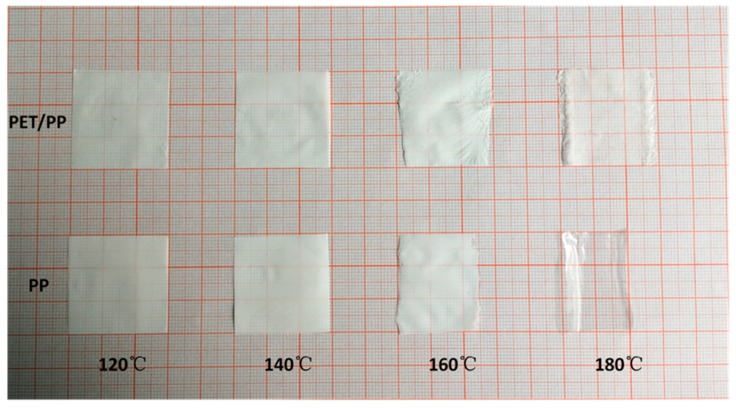
Photography of changes in the separator profile as a function of temperature.

To further illustrate the thermal shrinkage and the meltdown behavior of the composite separators, the relevant separators are sandwiched by two glasses and then placed in an oven at various temperature from 120 °C to 180 °C for 0.5 h. As shown in Figure 9, The PP separator shrank severely (33%) with the color changing from white to semi-transparent, whereas the composite separators changed little in dimension.

**Figure 10:**
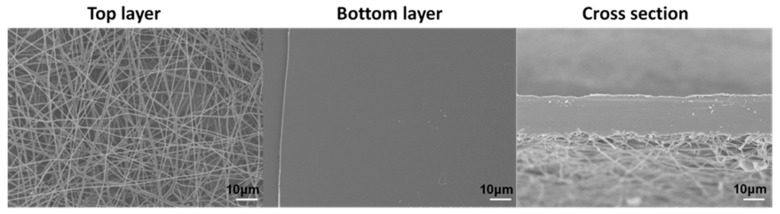
FE-SEM photograph for PET/PP-22% after shutdown.

To directly prove the thermal shutdown function of the separator, the PET/PP-22% was sandwiched by two glasses and placed in an oven at 170 °C for 0.5 h. The morphology of PET/PP-22% after the shutdown was characterized (Figure 10). It can be seen from the surfaces and cross-section that the PP separator as a base membrane melted and combined with the surface of the PET nonwoven membrane, turning the porous ionic conductive polymer film into a non-porous insulating layer between the electrodes. The closure of the pores prevented possible shortage of the battery and increased the safety performance of the battery. All composite separators present similar morphology with the same heating process, demonstrating the effective shutdown function of the composite separator.

### 3.3. Electrochemical Performance

**Figure 11:**
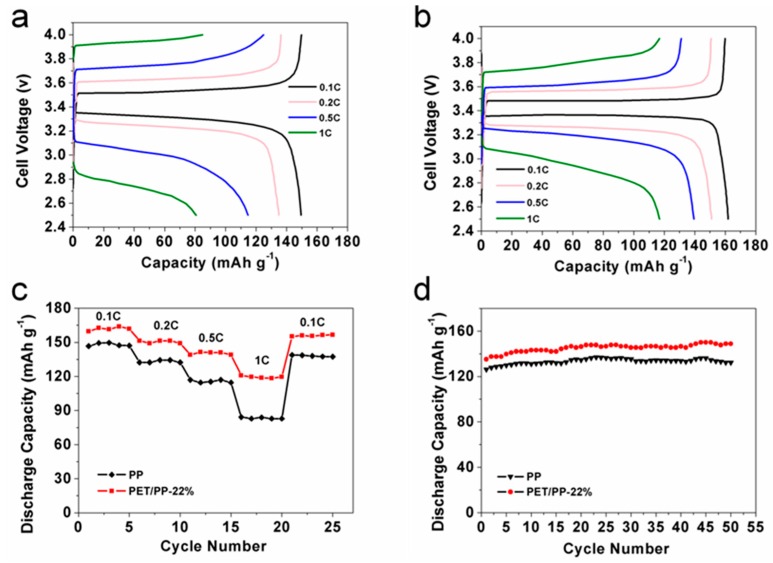
Charge and discharge profiles of cells assembled (**a**) PP and (**b**) PET/PP-22%; (**c**) rate capability of the cells with PP separator and PET/PP-22% at different C-rates (LE, 2.5–4.0 V); (**d**) Cycle performance of cells with PP separator and PET/PP-22%.

As PET/PP-22% showed the highest ionic conductivity and electrolyte uptake, it is expected that the separator could possess high battery performance. To demonstrate this hypothesis, the charge–discharge behaviors of the of the half-cell assembled with the PET/PP separator using the LiFePO_4_ cathode were investigated. Figure 11a,b revealed that the discharge capacity of the cells gradually decreased with the increase of discharge rate. The cells with PET/PP-22% achieved a high discharge capacity of 161 mAh g^−1^ at the discharge rate of 0.1 C; at 1 C, the cell kept 74% of the discharge capacity at 0.1 C, which was much higher than the cell with the PP separator at a relevant discharge rate. Furthermore, the rate performance of the separators was investigated (Figure 11c). The PET/PP-22% showed improved discharge capacity compared to the PP separator over the whole rate range investigated, demonstrating the high potential of PET/PP-22% for practical battery applications.

Figure 11d showed the cyclability of the PP separator and the PET/PP-22%. Both the PP separator and PET/PP-22% exhibited stable discharge capacity up to at least 50 cycles with little degradation. The cell based on PET/PP-22% had a discharge capacity of 149 mAh g^−1^ after 50 cycles, higher than 134 mAh g^−1^ for cell with PP separator. The improved discharge performance of the PET/PP-22% is believed to be contributed by its excellent electrolyte affinity. The excellent rate performance and cycling performance of PET/PP-22% indicates its high potential for practical battery applications. A list of performance parameters of the PET/PP-22% separator and their comparison with typical separators reported recently is shown in Table S1. From the comparison, it can be seen clearly that the PET/PP-22% separator exhibits a superior mechanical property and excellent electrochemical performance.

**Figure 12:**
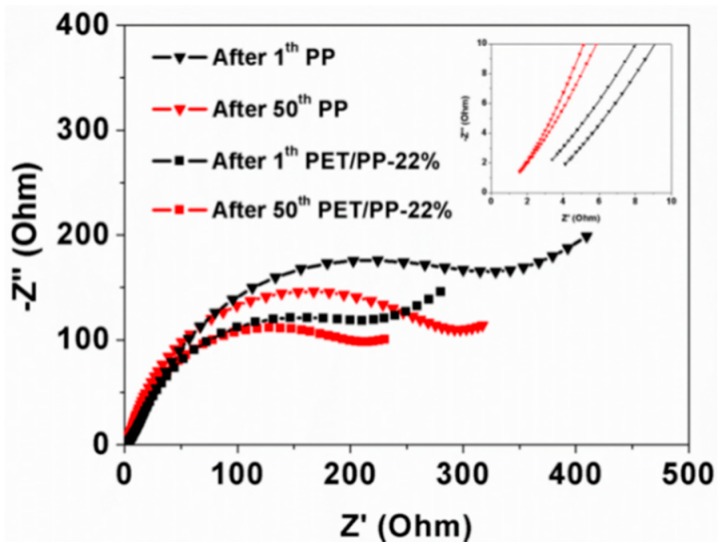
The variation in AC impedance spectra (1st cycle-50th) of cells assembled with PP separator and PET/PP-22%.

In order to further understand the excellent electrochemical performance of PET/PP-22%, the EIS spectra of the cells with PP or PET/PP-22% were recorded before and after charge–discharge cycles (Figure 12). The intercept of Nyquist curves at the Z’ axis indicates the combination resistance (R_Z_) which is related to the ionic resistance of the electrolyte. The semicircle in the high frequency area represents the resistance through the solid electrolyte interface (SEI) layers (R_SEI_) [36]. As determined from the Nyquist plots, the cell with PET/PP-22% had a lower R_Z_ after both 1st cycle (1.17 Ω) and 50th cycle (0.35 Ω) than the cell with the PP separator (1.88 Ω and 0.37 Ω, respectively). Furthermore, the R_ct_ of the cell with PET/PP-22% separator was also lower than the cell with the PP separator in all cases. The low R_z_ and R_ct_ of the cell based on PET/PP-22% could be ascribed to the higher porosity, electrolyte uptake, and better electrolyte wettability of the separator.

**Figure 13:**
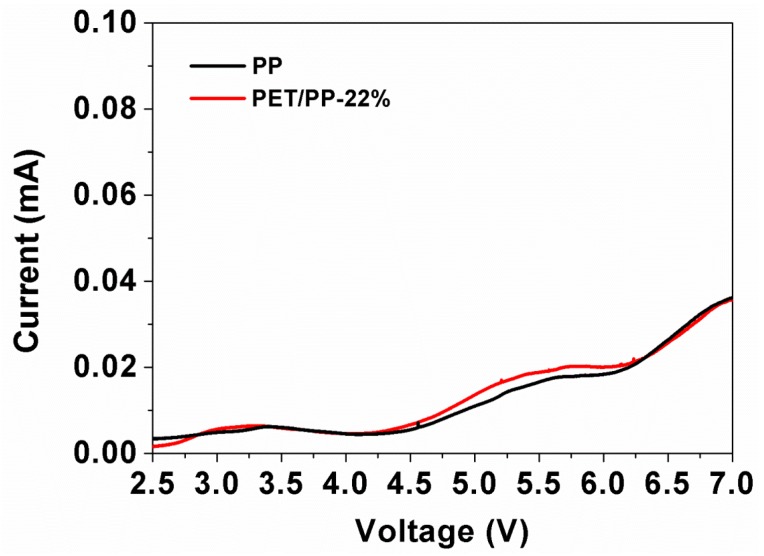
Liner sweep voltammetry of SS/PP/Li and SS/PET/PP-22%/Li cell.

Finally, the electrochemical stability window of the PP separator and the composite separator were evaluated by linear sweep voltammetry (Figure 13). A very low background current was measured below 4.5 V, followed by a small increase in current flow which indicated the onset of electrochemical decomposition of the electrolyte. Considering the fact that almost all of the commercial electrode materials have an electrode potential below 4.5 V (Vs. Li/Li^+^), the electrochemical stability of PP/PET-22% is high enough for commercial battery applications.

## 4. Conclusions

A facile electrospinning process, which can be easily scaled up for industrial production, is developed to prepare a composite PET/PP separator. A precursor solution with a concentration of 22% is identified as the optimal solution for the preparation of the separator. The optimized PET/PP separator exhibits excellent thermal stability with little dimensional change at a temperature up to 180 °C. In addition, the separator has a shutdown function, which could effectively improve the safety performance of the separator. The PET/PP separator also inherits excellent electrolyte affinity from the PET component and therefore shows improved electrochemical performance compared to the commercial PP separator. Our results show that the novel electronspun PET/PP separator can function as a competitive alternative to the existing polyolefin separator and may find enormous applications in different battery applications.

## Figures and Tables

**Table 1:** The thickness, measured resistance, and ionic conductivity of the separators.

Separator	Thickness/μm	Resistance/Ω ^1^	Ionic Conductivity ^1^/mS cm^−1^
PP	20	2.15	0.503
PP/PET-16%	29	3.17	0.495
PP/PET-18%	30	2.83	0.573
PP/PET-20%	31	2.52	0.665
PP/PET-22%	32	2.21	0.782

^1^ measured at 25 °C.

## Supplementary Materials

The following supplementary materials are available online at http://www.mdpi.com/2073-4360/10/6/574/s1. Table S1. Comparison of the properties of the PET/PP separator with recently reported separators.
