# Supplementary material for: Electrospun Polyethylene Terephthalate Nonwoven Reinforced Polypropylene Separator: Scalable Synthesis and Its Lithium Ion Battery Performance
Source: Polymers (Basel). 2018 May 23;10(6):574. doi: 10.3390/polym10060574 (PMC6403667; doi:10.3390/polym10060574)
Supplement: Supplementary file 1 [file polymers-10-00574-s001.pdf]

Article

Electrospun Polyethylene Terephthalate Nonwoven Reinforced Polypropylene Separator: Scalable Synthesis and Its Lithium Ion Battery Performance

Haopeng Cai [1], Xing Tong [1], Kai Chen [2], Yafei Shen [2], Jiashun Wu [1], Yinyu Xiang [3], Zhao Wang [1,4,*] and Junsheng Li [3,*]

1 School of Materials Science and Engineering, Wuhan University of Technology, Wuhan 430070, China; cai_haopeng@whut.edu.cn (H.C.); 18771994076@163.com (X.T.); whutwjs@163.com (J.W.)

2 Wuhan Jingce Electronic Technology Co., Ltd., Wuhan 430070, China; chenkai@wuhanjingce.com (K.C.); shenyafei@wuhanjingce.com (Y.S.)

3 School of Chemistry, Chemical Engineering and Life Sciences, Wuhan University of Technology, Wuhan 430070, China; 243869@whut.edu.cn (Y.X.)

4 State Key Laboratory of Advanced Technology for Materials Synthesis and Processing, Wuhan University of Technology, Wuhan 430070, China

* Correspondence: wangzhao2070@126.com (Z.W.); li_j@whut.edu.cn (J.L.); Tel.: +86-027-87756662 (J.L.)

Table S1. Comparison of the properties of the PET/PP separator with recently reported separators.

separator	thickness/ µm	thermal shrinkage/ %	tensile strengthen/ MPa	electrolyte	electrolyte uptake/%	conductivity/ mS cm^{-1}	anode/ cathode	1st cycle discharge capacity/ mAh g^{-1}	Thermal shutdown function
**PET/PP**	**32**	0 (120 °C, 1 h)	**85**	**1M $LiPF_6$ in EC:DMC:DEC**	**293**	**0.782**	Li/ $LiFePO_4$	163 mAh g^{-1} (0.1C)	yes
PVDF-CTFE coated PP[1]	~18	-	-	1M $LiPF_6$ in EC/DMC/EMC	1.3 mg/cm^2	0.75	Li/ $LiFePO_4$	89 (8 C)	no
PANI nanowire coated PI[2]	42	0 (200 °C, 2 h)	34	1M $LiPF_6$ in EC/DMC/EMC	610	2.33	Li/ $LiFePO_4$	133 (0.2 C); 83 (2 C); 11 (10 C)	no
SiO_2/Al_2O_3 coated PI[3]	40	-	-	1M $LiPF_6$ in EC/DMC/EMC	519	2.92	Li/ $LiFePO_4$	136 (0.2 C); 117 (2 C);	no
silica grafted PE[4]	8	20 (150 °C, 0.5 h)	14.46	1M $LiPF_6$ in EC/DMC/EMC (1/1/1, v/v/v)	-	0.45	Li/ $LiFePO_4$	-	no
heat treated PVDF[5]	-	-	9.5	1M $LiPF_6$ in EC/DEC (1/1 v/v)	~380	1.35	Li /$LiFePO_4$	162.3 (0.2)	no
Al_2O_3 nanowire[6]	~50	0 (150 °C, 1 h)	-	1M $LiPF_6$ in EC/DMC/DEC (4/4/2, v/v/v)	190	1.7	Li /$LiFePO_4$	110 (2 C)	no
magnesium aluminate[7]	30-50	0 (120 °C)	1.5	1M $LiPF_6$ in EC/DEC (1/1, w/w)	81	1	Li /$LiFePO_4$	80 (1 C)	no
MMT[8]	70-80	<0.1 (125 °C, 1 h)	-	1M $LiPF_6$ in EC/DEC (1/1, w/w)	251	0.901	Li /$LiFePO_4$	105 (0.1 C)	no

References:

1. J Solid State Electr. 2014;18(9):2451-2458.
2. J Power Sources. 2015;299:417-424.
3. J Membrane Sci. 2015;493:1-7.
4. ACS Appl Mat Interfaces 2015;7(43):24119-24126.
5. J Membrane Sci. 2016;504:97-103.
6. ACS Appl Mat Interfaces 2015;7(1):738-742.
7. J Membrane Sci. 2014;471:103-109.
8. Ionics. 2014;20(7):943-948.
